# Flavonoids Effects on Hepatocellular Carcinoma in Murine Models: A Systematic Review

**DOI:** 10.1155/2018/6328970

**Published:** 2018-02-28

**Authors:** Estefanny Ruiz García, Eliana Alviárez Gutierrez, Fabiana Cristina Silveira Alves de Melo, Rômulo Dias Novaes, Reggiani Vilela Gonçalves

**Affiliations:** ^1^Department of Animal Biology, Federal University of Viçosa, 36570-900 Viçosa, MG, Brazil; ^2^Department of Food Science and Technology, Federal University of Viçosa, 36570-900 Viçosa, MG, Brazil; ^3^Institute of Biomedical Science, Department of Structural Biology, Federal University of Alfenas, Alfenas, MG, Brazil

## Abstract

The hepatocellular carcinoma (HCC) is the second most common cause of cancer deaths worldwide. It occurs primarily as manifestation of other pathological processes, such as viral hepatitis, cirrhosis, and toxin exposure that affect directly the cellular process. Studies were selected from PubMed and Scopus databases according to the PRISMA statement. The research filters were constructed using three parameters: flavonoids, hepatocellular carcinoma, and animal model. The bias analysis of the 34 selected works was done using the ARRIVE guidelines. The most widely used flavonoid in the studies was epigallocatechin gallate extracted from green tea. In general, the treatment with different flavonoids presented inhibition of tumor growth and antiangiogenic, antimetastatic, antioxidant, and anti-inflammatory activities. The bias analysis evidenced the absence of methodological processes in all studies, such as the age or weight of the animals, the method of flavonoids' extraction, or the experimental designs, analytical methods, and outcome measures. It has been known that flavonoids have a protective effect against HCC. However, the absence or incomplete characterization of the animal models, treatment protocols, and phytochemical and toxicity analyses impaired the internal validity of the individual studies, making it difficult to determine the effectiveness of plant-derived products in the treatment of HCC.

## 1. Introduction

The hepatocellular carcinoma (HCC) is a widely distributed type of cancer with high prevalence, being the second most common cause of cancer deaths worldwide. It is responsible for high mortality rates, with approximately 745,000 annual deaths, according to world the cancer report in 2014 [1]. HCC occurs primarily as a manifestation of other pathological processes, such as viral hepatitis, cirrhosis, and toxin exposure, which affect directly the liver, increasing inflammatory processes and leading to necrosis and accumulation of the connective tissue [2].

The viral hepatitis B (VHB) and viral hepatitis C (VHC) are considered risk factors in the HCC [1], causing alteration by modification in the cell environment and producing genetic pathways instability. One of the pathways affected in the infection process is the P53 protein. This pathway is one of the main regulators of the cell cycle, which is suppressed by the HBx gene expression in the viral hepatitis infection [2]. P53 protein is affected by the action of other factors, such as aflatoxin exposure, which is a toxin produced by the fungus* Aspergillus* spp. The cytochrome P450 converts this toxin into exo-8,9 epoxide, which is able to affect this gene, damaging the DNA [3].

The Wnt/catenin signaling pathway is involved in liver development, amino acid metabolism, and oxidative stress, also acting in the HCC progression by the mutation of *β*-catenin and inactivation of the negative regulation of the APC/Axin1/GS3KB complex over Wnt/catenin. This process leads to the transcription of different genes involved in cell proliferation [4]. Meng et al. [5] demonstrated that MAP kinases and PI3K/AKt/mTor pathways are affected in HCC by the action of CAMK2^*γ*^, causing hyperproliferation of hepatocytes. Other studies demonstrated that IL-6 in HCC is overproduced in malignant transformation of the hepatic tissue [6, 7].

Conventional drugs have shown a toxic effect in the liver, thymus, and spleen of patients with HCC and have not also been effective due to the resistance of cancer cells [8]. Currently, there is growing interest in the study of metabolites from plants that have presented beneficial effects to human health. These components have effects on disease prevention for its antioxidant activity, anti-inflammatory, and anticancer potential [9]. Phenolic compounds of plants, mainly flavonoids, are known by its beneficial effects in treatments for various diseases. Many studies have suggested chemopreventive effects of flavonoids on different cancer types, including HCC. They can act regulating several pathways associated with the progression of HCC [8]. Flavonoids are heterogeneous polyphenols found in a wide variety of plants, mainly in fruits and vegetables. The flavonoid structure has two aromatic rings, A and B, joined by a 3-carbon bridge, usually in the form of a heterocyclic ring, C [10]. The variations in substitution patterns to ring C result in more flavonoids classes, like flavonols, flavones, flavanones, flavanols (or catechins), flavan-3,4-diol, isoflavones, and anthocyanidins [11]. These metabolites have been effectively providing protection and enhancing the repair process through the control of different molecular pathways, which regulates the expression and suppression of genes, protein activities, and the cell cycle [2, 12]. The flavonoids have shown to protect the cell against cancer progression by the activation of proapoptotic and antiproliferative pathways, or inactivation of the antiapoptotic pathways, such as the inactivation of ERK/2 and inhibition of proteins like MMP2 [8, 13] and COX-2 gene [14], which are widely expressed in tumor cells.

Some clinical and preclinical studies have tried to demonstrate the positive effect of plant compounds and their derivatives in the treatment of HCC. However, this hypothesis is not always confirmed, mainly due to the large methodological variations involving the obtaining of the compounds, the therapeutic schemes, and the mechanisms of action. Therefore, it is critical to compile data from various studies in order to clarify the aforementioned discrepancies. In this context, the systematic review, although little used in animal studies, is a powerful tool that incorporates the variability among the studies and allows the obtaining of an overall estimate of the use of plant extracts in the treatment of HCC in murine models. Besides this systematically reviewing, the preclinical evidence in an objective manner (unlike the widely used narrative reviews) has never been carried out before and might provide us with reliable and solid new evidence on whether or not plant extracts and its derivatives could be beneficial in the treatment of HCC. Based on this, our systematic review was developed to determine if there is a rational basis in the selection of all plant species and subclasses of investigated flavonoids. In addition, we performed a critical analysis of preclinical studies in order to improve the quality of the reports and thus prevent the reproduction of methodological failures that could compromise the development of clinical studies.

## 2. Material and Methods

The systematic review adhered to PRISMA (Preferred Reporting Items for Systematic Reviews and Meta-Analysis) [49] guidelines, including search strategy, selection criteria, data extraction, and data analysis.

### 2.1. Literature Research

The papers analyzed in this review were selected from two electronic databases, PubMed (https://www.ncbi.nlm.nih.gov/pubmed) and Scopus (https://www.scopus.com/freelookup/form/author.uri), completed in October 05, 2015, at 12:31 p.m. The keywords used were based on filters constructed by three criteria: flavonoids, hepatocellular carcinoma, and animal model. The strategy used in the construction of the filters on PubMed platform was the hierarchical distribution of the MESH terms. In the Scopus database, a standardized filter for animal studies was applied, and the same PubMed search strategy was adapted and used on it [50] (Table S1). Only experimental studies published in English were included. Reviews, comments, and notes, as well as unpublished studies, were not considered. The studies were selected based on the inclusion criteria mentioned below:

- Studies showing the effect of flavonoids in the HCC in murine model for animal experimentation were included.
- Studies only* in vitro*, performed in humans and using synthetic flavonoids, were excluded.
- Studies reporting the use of flavonoids in the treatment of other tumors in organs other than the liver were also withdrawn.

Exception was done with works that used commercial green tea extracts or synthetic flavonoids derived from green tea, which was included for the commercial value of the plant. We searched the references of the select studies for others studies that met the inclusion criteria.

### 2.2. Extraction and Data Management

Abstract selection: three independent reviewers (ERG, RDN, and RVG) selected eligible studies based on title and abstract analysis. In case of disagreements a fourth reviewer (FCSAM) decided whether the study met the inclusion and exclusion criteria. In order to discard subjectivity in the data collection and selection process, the information was independently extracted by the two reviewers (ERG, EAG) and analyzed separately. Data from each study were extracted and tabulated using standard information, such as features of the publication (author, year, country, and title); plant (plant family and species, used part, flavonoid type, and extraction and purification method);* in vitro* assay; experimental model (animal model, strain, sex, age, body weight, administration, and frequency of treatment); induction of hepatocellular carcinoma/concentration and volume; tumor measurement;* in vivo *analysis (Table 1). When there was difficulty in extracting the data or obtaining the studies, the authors were contacted by e-mail to provide the necessary information. Subsequently, the data were compared and the conflicting information were identified and corrected by discussion and consensus among the reviewers.

### 2.3. ARRIVE Analysis

The quality of the articles was analyzed according to the criteria described on Animal Research: Reporting of* In Vivo* Experiments (ARRIVE). These criteria are based on brief descriptions of essential characteristics of all studies using animal models, such as theoretical and methodological basis, research objective and improvement of analytical methods, statistical design, sample calculations, and outcome measures [51]. Considering that this systematic review aims to evaluate important aspects of the referenced publications, a table summarizing all the investigated aspects was built, as well as their relevance, describing positive and negative aspects of the recovered studies.

## 3. Results

### 3.1. PRISMA Guideline

**Figure 1:**
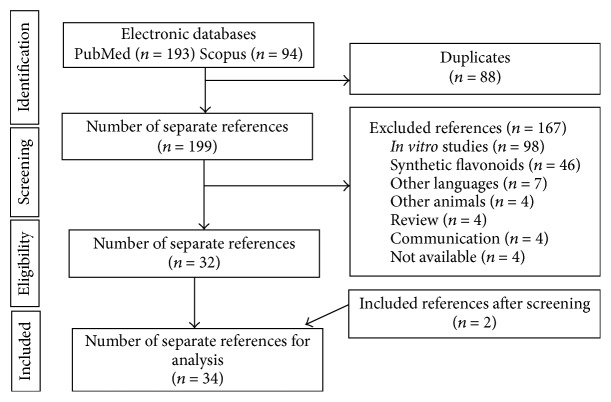
Flow diagram of the systematic review literature search results. Based on “Preferred Reporting Items for Systematic Reviews and Meta-Analyses: The PRISMA Statement.” http://www.prisma-statement.org. From: Moher D, Liberati A, Tetzlaff J, Altman DG, The PRISMA Group (2009). Preferred Reporting Items for Systematic Reviews and Meta-Analyses: The PRISMA Statement. PLoS Med 6(6): e1000097. doi:10.1371/journal.pmed1000097. For more information, visit http://www.prisma-statement.org/.

The PRISMA diagram illustrates the selection process of the studies (Figure 1). The initial search resulted in 287 studies (193 on PubMed and 94 on Scopus), out of which 88 were duplicates. After reading the title and abstract, 167 studies were excluded, since they addressed subjects not related to the chosen topic. Among the excluded studies, we can highlight 98 about* in vitro* studies, 46 studies related to the use of synthetic flavonoids, and 23 excluded by other criteria listed in Figure 1. After analysis of the eligibility criteria, 32 studies were included in this review. The references' list of the 32 articles selected was analyzed and 2 of them fitted the inclusion criteria, totalizing 34 studies.

### 3.2. Data Extraction

**Figure 2:**
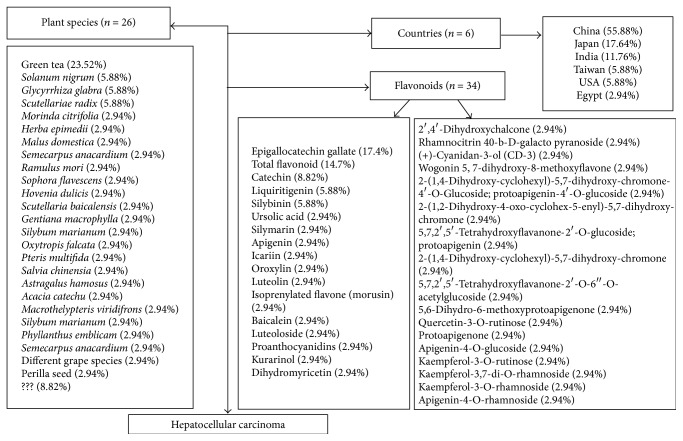
Schematic representation of plant species, used flavonoids, and countries with researches about benefits of flavonoids in hepatocellular carcinoma.

The selected studies were conducted in 6 different countries, mainly China (*n* = 19, 55.88%), followed by Japan (*n* = 6; 17.64%), India (*n* = 4; 11.76%), Taiwan (*n* = 2; 5.88%), USA (*n* = 2; 5.88%), and Egypt (*n* = 1; 2.94%). The number of plant species was 26 and 34 flavonoids were extracted and studied. The most widely used flavonoid was epigallocatechin gallate (EGCG) (17.64%) extracted from green tea, followed by total flavonoid (14.7%), catechin (8.82%), liquiritigenin (LQ) extracted from* G. radix *(5.88%), and Silibinin (5.88%) extracted from* S. marianum *(Figure 2). Five studies used the whole plant (14.7%), other studies extracted flavonoids from the leaf (8.82%), root, and seed (5.88%), and different studies used the radix, fruit, nut, and heartwood (2.94% each). Seventeen studies did not specify the used part of the plant to obtain the flavonoid (49.98%) (Table 1).

Considering the animal strain, most studies used mice of the BALB/c strain (*n* = 10; 29.41%), Kunming (*n* = 6; 17.64%), followed by ICR (*n* = 4; 11.76%). Other studies used Wistar rats (*n* = 2; 5.88%), Donryu rats (*n* = 2; 5.88%), and Sprague Dawley and B6C3F1 rats (*n* = 1; 2.94% each), and 1 study did not specify the rat strain (2.94%). Most studies described the sex of the animals (*n* = 28; 82.35%), where 24 of the works used males (70.58%), 2 used females (5.88%), and other 2 works used males and females (5.88%). Six studies did not specify the sex of the animals (17.64%). The age and the weight of the animals were specified in 9 studies (26.47%), 10 studies only described the age of the animals (29.41%), 10 studies only described the weight of the animals (29.41%), and 5 works did not specify the weight of the animals (14.70%) (Table 1).

The process of carcinoma induction was determined in all studies. The induction of HCC by transplanted cancer cell lines was made in 27 studies (79.41%), 3 studies induced by the administration of N-nitrosodiethylamine (NDEA) (8.82%), 2 studies by the administration of aflatoxin B1 (AFB1) (5.88%), 1 study by the administration of thioacetamide (2.94%), and others by spontaneous hepatocarcinogenesis (2.94%). The main transplanted cell lines by the induction were H22, with 10 studies (29.41%), HepG2 with 4 studies (11.76%), Huh7 and SMMC7721 with 3 studies each (8.82%), and AH109A with 2 studies (5.88%) (Table 1).

**Figure 3:**
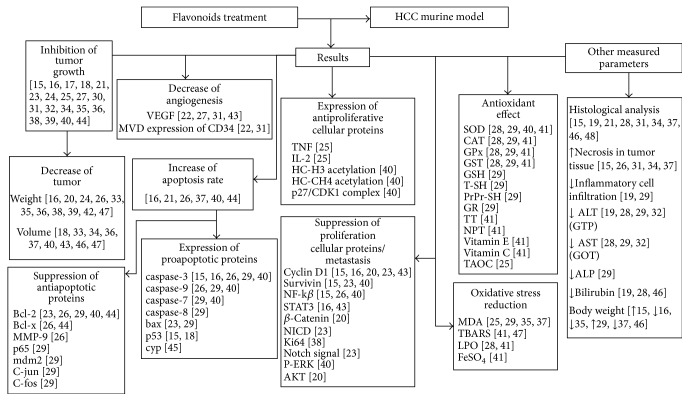
Main results demonstrating the action of flavonoids in the treatment of hepatocellular carcinoma in murine models.

**Figure 4:**
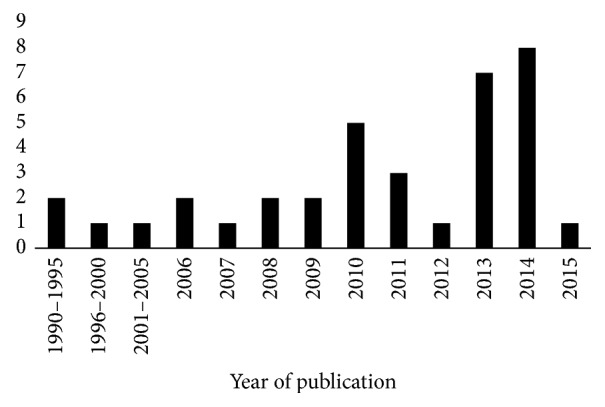
Number of papers published by worldwide scientists using flavonoids in the treatment of hepatocellular carcinoma in the last 25 years (between 1990 and 2015).

The treatment with flavonoids in the animal models studied (rats and mice) was shown to be efficient in decreasing the size and volume of tumors by the inhibition of tumor growth, suppression of antiapoptotic proteins expression, and increase in proapoptotic proteins expression. Proteins that favor cell proliferation and the process of metastasis were inhibited, and proteins that disrupt the process of cell proliferation were expressed in greater quantity in the treatment groups, when compared to the control groups of the different works. A decrease in angiogenesis was also reported, and an antioxidant effect was evidenced mainly with the decrease of markers of oxidative stress. Other parameters, such as the presence of necrosis in the tumor tissue, inflammatory infiltrate, and blood markers were analyzed (Figure 3). The results of our work suggest an increase in the interest for products of plant origin in recent years, mainly due to the use of flavonoids in the treatment of HCC in 2013 and 2014, when compared to 1990–2009 (Figure 4).

### 3.3. ARRIVE Analysis

From the articles analyzed in this systematic review, 64.71% reported the permission from the ethics committee for performing the research. Regarding animal experiment, 61.76% specified the total number of animals used in each experiment, 82.35% specified the number of experimental and control groups, and 44.11% reported the number of animals in each group included in the analysis. No study reported if any animals or data were not included in the analysis or how the number of animals was decided, while 17.64% of the works described that the results can be interpolated to other species, such as humans. 55.88% of the studies specified how the animals were allocated to experimental groups, and 14.70% indicated the characteristics and health status of the animals before the treatment. The origin of the animals was specified in 85.29% of the papers, where 64.71% specified the sex of the animals, 52.94% reported the weight, and all studies specified the animal species. The developmental stage was reported in 50% of the works, 58.82% indicated any steps taken to minimize the effects of subjective bias when allocating animals to treatment, 41.17% specified the housing of the animals, and 50% reported the husbandry conditions. Regarding the statistical methods, 70.58% of the studies provided details of the statistical methods used for each analysis and specified the unit of analysis for each dataset. The modifications made to reduce adverse events in the experimental protocols were specified only in 2.94% of the studies (Table 2).

## 4. Discussion

In this study we conducted a systematic review to describe the main findings in the literature regarding flavonoids effects in the treatment of HCC in murine models. Despite the great heterogeneity of the studies it is possible to conclude that, in general, the use of flavonoids is effective in the treatment of HCC. The option for plant research is due to the fact that they are the oldest and most important source of bioactive compounds for the treatment of several diseases and still contribute directly to the development of new drugs [52, 53]. The results of our work suggest an increase in the interest for products of plant origin in recent years, mainly natural products in treatment of HCC.

The use of natural products for the treatment of different tissue changes is an old practice, and its use is closest to popular utilization. It is noteworthy that the biological activity of a natural product is often due to the synergism between its constituents, which potentiates the therapeutic properties [54]. Therefore, natural products therapy represents a promising alternative for the treatment of cancer, especially HCC, which is classified as an extremely aggressive type of cancer with high morbidity and mortality worldwide [1]. Our findings indicated that although there are punctual research initiatives in developed countries, the search for new treatment options for HCC using plant-derived natural resources is a concern of developing countries. Well-defined geographic patterns were expected, in which research initiatives on a particular disease concentrate in regions that have tradition in research using natural products, such as China, Japan, and India. This type of observation was possible because the systematic review allows evaluating, from multiple studies, the variability of individual works, allowing the establishment a global estimate on the use of flavonoids in the treatment of HCC [55]. However, although systematic review papers present a high level of scientific evidence, and the selection process is based on widely accepted practices, the results presented here should be interpreted with caution. This is related to the selection process of studies that may be biased due to different factors, such as initial exclusion based on just reading the titles and abstracts, or the inclusion of more than one study of the same group of researchers. Even so, the results presented will serve as a basis for a critical analysis of the main findings that demonstrate the effect of plant-derived flavonoids on HCC treatment, since it is the first systematic review work to study the effect of these compounds on HCC.

In this review, the research has taken into account works carried out with murine models, which are widely used as a model for the development of human diseases. Several researches have reported the ease in handling these animals, as well as their similarity to humans regarding the metabolic and molecular processes that occur in different diseases, including cancer [56, 57]. In the reviewed studies, most of the investigations used mice from different strains, mainly BALB/c nude as a model for the induction of HCC. Besides this, genetic engineering has advanced in the genotypic modification of mice and rats. Strains of nude mice provide a greater development of the carcinogenic process because they favor the appearance of spontaneous tumors and provide a more stable biochemical environment [58, 59]. Athymic mice also have advantage when the transplantation of tumors is present, as this prevents immunological rejection [60]. Moreover, in addition to the decrease in maintenance time, the experimental processes with mice, compared to rats, also decrease the time of tumors induction [61]. Despite the percentage of used rats, this murine is also a good model for the induction of HCC, since different strains present appropriate characteristics for cancer induction, such as increased neovascularization, predisposition to metastasis, and rapid development of the tumor [62]. However, it presents limitations, such as the low availability of genetically knock out rats of specific genes [63].

In these investigations, there was a strong tendency to use male murine, and the percentage of researches that used females or the two genders was low. This is probably because males have been shown to be more predisposed to HCC, and this is attributed to the increase of IL-6 concentrations released by Kupffer cells in response to hepatic stress [64]. That does not occur in the same way in females due to the action of estrogens that inhibit the action of IL-6, disrupting the activity of NF-*κ*B and other transcript factors [65].

The induction process of HCC in most investigations is performed by the transplantation of cell lines and has been shown to be effective in murine model for HCC, especially in mice [63, 66, 67]. The use of xenograft models is widely used due to the rapidity with which the tumor is established and the carcinogenic process is developed [60]. In the case of reproducibility of metastases, the use of orthotopic models has given better results. In the study made by Fan et al. [21], the observation of the metastatic process in the lung is performed by means of an injection of HCC cells into the caudal vein of the animal, the induction of metastasis in the lung being effective. Miura et al. [42], on the other hand, performed the induction of hepatocarcinoma by subcutaneous injection and, subsequently, observed the metastatic process in lungs and inguinal and axillary lymphatic nodes. Several authors suggest that the use of orthotopic models for the visualization of metastatic processes has more effective and reproducible results in the case of HCC with transplants of HCC cells directly in the murine liver [62, 63, 68]. Other induction methods were performed using toxic components as NDEA, aflatoxin B1 (AFB1), and thioacetamide, which are reported to have an action in the instability of different pathways involving the cellular cycle control [1]. Investigations on these toxic agents are important to study the damage caused by them. AFB's damage, for example, is directly related to the chromosomal damage and DNA degeneration [69]. Works of this type give a more specific approach to the problems that occur in countries such as China and Africa, where the prevalence of people affected by pollution with AFB is still high [70].

All models used by the researchers showed efficiency on the carcinoma induction, with physiological changes in the animals and tumor growth. However, a murine model is far from the human condition, since these animals present a high mortality rate and frequently do not develop the chronic disease. Although canine and primate models are indicated as the most closed related to human disease [71], the limited availability of dogs and monkeys, the high cost of maintenance in large animal facilities, and ethical issues related to animal welfare hamper the widespread of these models [72].

In our study, the parameters evaluated in the HCC development in murine models showed mainly the inhibition of tumor growth and physiological changes in the animals treated with flavonoids when compared to control groups. Flavonoids such as Wogonin stopped tumor growth with an inhibition of 65% when associated with a drug commercially used in the treatment of HCC [30]. He et al. [73] reported the antiproliferative activity of Wogonin by the control of the oncoprotein CDK8 involved in the proliferation process in colorectal cancer and by acting on the Wnt/b-catenin pathway. Flavonoids have also been shown to have an effect on reducing the size and weight of different types of tumors [74, 75]. Reducing tumor size was reported in five studies, in which the flavonoid CD-3 showed beneficial effects both in* in vivo* and* in vitro* analyzes [29], due to the activation of caspases leading to an increase in the apoptotic pathway. The tumor weight was measured in 6 studies, where in most treatments a decrease in a dose dependent manner was observed. The flavonoid Baicalein [20] showed a reduction of 78% of the tumor weight. Other studies reported that Baicalein induced apoptosis in human gastric carcinoma, breast cancer, and hepatocellular carcinoma line cell when combined with the alkaloid 10-hydroxycamptothecin, usually used alone in cancer treatment [76]. This result can suggest that the apoptotic process is related to the suppression of tumor growth and reduction of tumor weight.

In addition to the morphological factors, the activation or inactivation of molecular pathways that halt the progression of the disease is of great importance in this type of research. The expression levels of proteins and growth factors in tumor tissue are an important factor that showed the progression of the carcinogenesis. In general, the use of flavonoids promoted decrease of VEGF and micro vesicular density of vessels and showed a decrease in angiogenic markers CD31, supporting the findings of other studies that have demonstrated reduced vascularization in metastasis processes in lung and kidneys tumors [67]. Associated with vascular increase, tumor cells acquire the property of evading apoptotic signals and show increased activity to promitotic signals [77]. These characteristics lead to uncontrolled cell division and decreased apoptosis, which results in rapid tumor growth [78]. An effective therapeutic agent for controlling tumor growth should be able to activate the proapoptotic signaling pathways and/or inhibit the antiapoptotic pathways. In addition, it must be able to control cell division by modulating the cell cycle; for this, it must be effective in controlling important cellular pathways. One of the central components in the regulation of apoptosis and the cell cycle is NF-k*β*, an important factor that induces the transcription of several antiapoptotic proteins and promotes cell replication [78, 79]. Among the main apoptotic pathways, we can highlight the caspase pathway, which is responsible for a series of processes that cleave the DNA and the cytoskeleton [80], altering the permeability of the mitochondrial membrane [79]. In this systematic review, the studies have shown that, in general, flavonoids treatment activates proapoptotic pathways and decreases cell replication, which results in decreased tumor growth. These findings show the therapeutic potential that flavonoids represent for the treatment of cancer.

Another important signaling pathway in cell survival is MAP kinases proteins, which have a regulatory role in proliferation, differentiation, and cell migration and has an overexpression in carcinogenic and metastatic processes [81]. The flavonoids used in the experiments selected had a control over this pathway, inhibiting the proliferation of cancerous cells. One of the major proteins in the activation cascade MAPKs are ERKs that can be activated by reactive oxygen species (ROS) and induce activation of cell survival signals, promoting cell proliferation [82]. The enzyme threonine kinase (AKT), another cell survival pathway, has been extensively studied because of its action on inhibition and blocking of protein-related activation of apoptosis [83]. AKT also blocks pathways that induce cell death under stress and is responsible for mediating activation complex IPK3/AKT, which regulates the IKK activation on the cascade for the degradation of IKB that will again lead to the activation of NF-k*β* [26, 30]. Baicalein, Wogonin, and Silibinin were shown to have an effect over the expression and activation of AKT, promoting the activation of apoptotic pathways [20, 30, 40]. Proteins like BAX and BAK, with proapoptotic activity, were upregulated in the tissue and showed suppressed expression of antiapoptotic proteins in culture tumor cells when treated with silibinin flavonoid (SIL) [23]. These targets are important in cancer therapy and the findings of this work showed that the use of flavonoids inhibits cell survival important pathways, such as MAP kinases, and IPK3/AKT, while it also favors the proapoptotic action of BAX/BAK proteins by acting positively on regulation of the mechanisms involved in cell survival and proliferation. In addition to the pathways mentioned above, other markers of cellular proliferation and formation of metastases were analyzed in the papers included in this review. Among them, we highlight cyclin D1, Notch signal, and *β*-catenin. These pathways appear to act individually or be in association with localized tumor development and metastasis formation [15, 20, 23].

Other important targets related to the anticancer therapy are the control of the cell cycle by the recognition of arrest points, where protein families such as cyclin regulated other proteins like CDKs and controlled the cellular cycle progress [84]. One of the most studied is the cyclin D1, which promotes the transition from G1 to S and is regulated by the GSK activation protein [85]. In our review, the use of flavonoids showed a positive effect in the treatment of HCC, since it inhibits the progression of the cell cycle. Baicalein [20],* Silybum marianum *[23], 2′,4′-dihydroxychalcone, and Luteolin [43] flavonoids showed inhibition on the activity of cyclin D1, suggesting cell cycle arrest and representing a promising therapy in the regulation of cell cycle. Some studies also measured the activation of proapoptotic factors on HCC, including the family of Ps. P38 proteins are activated for ASK, TNF receptor, and JNK that together promote apoptosis [86]. P53 proteins are important in the activation of metabolic pathway genes like CYP, a fundamental gene in the metabolism of lipids and sex steroids, and are known by their important activity in the cell cycle arrest, promoting genetic stability and control over DNA damage [87]. Mutations in the P53 gene are found in early cases of HCC in patients affected by AFB [88]. The results found in our review showed that P53 was upregulated in the cell culture of HCC treated with Dihydromyricetin [18], Cyanidan-3-ol [29], and Oroxylin [39] flavonoids, suggesting an induction of apoptosis in cancer cell lines, which would lead to the elimination of the tumor.

Flavonoids have been shown to exhibit high antioxidant activity, being effective in the protection of tissues from damage caused by ROS [89, 90]. In the reviewed articles, flavonoids like Silibinin were shown to be effective in reducing tumor in xenografts mice, acting on p-ERK activation, and reducing their activity [40]. Other flavonoids showed strong antioxidant activity, reducing stress oxidative markers like MDA [19, 25, 29, 31, 35] and stimulating the increase of antioxidant enzymes such as SOD [19, 28, 29, 31, 41].

Tumor metastasis encompasses a series of interrelated phenomena consisting of invasion and metastatic colonization, where malignant cells spread from primary tumor to organs [91, 92]. In this context, angiogenesis is a prerequisite for advancing the tumorigenic process, increasing the vascularization, allowing the tumor nurturing and the migration of cancer cells, favoring metastasis [93]. Involved in this process are enzymes and proteins that allow the development production of blood vessels, such as growth factors and metalloproteinases (MMPs) [33]. One of the MMPs involved in this process is the MMP9, which together with Symdecam 1 and FGF-2 regulates leukocyte cell migration during inflammation and tissue regeneration in wound healing, but in the oncogenic process, they are released by invasive cells, which degrade the extracellular matrix and favor cell migration and consequently the formation of metastasis [5]. The results of this work showed that all these markers are increased in the presence of metastases and have shown reductions in the treatment with flavonoids [19]. It shows that flavonoids protect the extracellular matrix from degradation, hindering the migration of tumor cells, delaying the invasion of capillaries, and, consequently, decreasing the formation of metastases and invasion of other tissues.

### 4.1. Limitations

Although our systematic review represents a proposal to group and critically analyze the evidence on the applicability of plant derivatives in the treatment of HCC, results' interpretation should consider some limitations. Our sampling frame was based on a specific number of databases. Thus, some articles may be not recovered due to the boundaries applied in the search strategy, as well as limitations in algorithms adopted in the search interfaces of each database. These aspects affect directly the sensitivity and specificity of the search strategy, which may have contributed to identify key articles. We attempted to mitigate these limitations by screening the reference lists of all articles, which are not limited to databases or any keywords-based search model. The relevant number of papers additionally recovered indicates the utility of this approach in a heterogeneous area, such as the evaluation of plant extracts in the treatment of HCC.

## 5. Conclusion

In general, flavonoids are effective in the treatment of HCC due to their capacity to reduce the tumor growth and inhibit the metastatic and angiogenic processes. Its repressive action of tumor responses occurs in different metabolic pathways. As a consequence, modulation of the carcinogenic signaling pathways reduces disordered replication, evasion of apoptosis, tissue invasion, and formation of metastasis, angiogenesis, and inflammation. However, the relevance of studies using flavonoids in the treatment of HCC is hampered by the lack of methodological rigor. Absence or incomplete characterization of the animal models, experimental groups, treatment protocols, phytochemical screening, and toxicity analysis of the plant products impairs the internal validity of the individual animal studies. Together with these limitations, contradictory results based on heterogeneous studies of the same plant species compromise the external validity of the evidence, making it difficult to translate animal data into clinical practice, as well as the relevance of the plant species as potential biotechnological targets in the development of new drugs to treat HCC. We believe that, in order to reduce the margin of error, the results should correlate* in vivo* and* in vitro* studies, since* in vitro* experiments are important for the identification of molecular markers to understand the action of the flavonoids in cancer treatment* in vivo*. Taking into account the fact that poor reporting quality does not always reflect the quality of the research actually carried out, we hope that our critical analysis may help to streamline preclinical researches in order to reduce methodological bias, improving data reliability and generalizability.

## Figures and Tables

**Table 1:** Description of the main characteristics of the studies using flavonoids in the treatment of hepatocellular carcinoma in murine models.

Ref.	Country	Article	Plant family/species	Used part of plant	Flavonoids type	Extraction and purification method	*In vitro *assay	Animal model/strain	Sex	Age/weight	Administration/ frequency	Induction of carcinoma concentration/ volume	Tumor measurement/ frequency	*In vivo* analysis
Wan et al., 2014 [15]	China	Preparation of morusin from *Ramulus mori* and its effects on mice with transplanted H22 hepatocarcinoma	Moraceae/*Ramulus mori*	Root bark	Isoprenylated flavone (morusin)	Ethanol extraction/Sephadex gel LH-20 and RF-HPLC	?	Mice/SPF ICR	♂	3–5 weeks/18–20 g	Intraperitoneal/ daily for 10 days	Transplanted H22 cells 1 × 10^7^ cells/ml/0.1 ml	Weight of tumor/2 weeks	H&E, qRT-PCR (P53, survivin, cyclin D1, caspase-3, NF-K*β*)

Shu et al., 2014 [16]	China	Kurarinol induces hepatocellular carcinoma cell apoptosis through suppressing cellular signal transducer and activator of transcription 3 signaling	Leguminosae/ *Sophora flavescens*	Root	Kushenol H, kurarinol, norkurarinol, kushenol N, kurarinone	Ethanol extraction, HPLC and Sephadex LH-20 column	HepG2, Huh-7, Bel-7402 cells humans, HL-7702, H22 cells mouse	Mice/Kunming	♂	?/18–22 g	Intraperitoneal/ daily for 10 days	Transplant of H22 cells 1 × 10^6^ cells/ml/?	Weight of tumor/experiment end	Serum (white blood, red blood cells, platelets, ALT, AST, BUN, uric acid, and CK), TUNEL, immunohistochemistry (STAT3)

Wang et al., 2014 [17]	China	Liquiritigenin induces tumor cell death through mitogen-activated protein kinase- (MPAKs-) mediated pathway in hepatocellular carcinoma cells	?/*Glycyrrhiza radix*	?	Liquiritigenin (LQ)	Purchased	PLC/PRF/5, HepG2 human cells	Mice/BALB/cA nude	♂	5 weeks/?	Intraperitoneal/ daily for 18 days	Transplanted PLC/PRF/5 5 × 10^7^ cells/ml/0.1 ml	Volume and diameters of tumor (mm3), body weight/every day	?

Zhang et al., 2014 [18]	China	Dihydromyricetin promotes hepatocellular carcinoma regression via a p53 activation-dependent mechanism	?/*Hovenia dulcis*	?	Dihydromyricetin (DHM)	Purchased	HepG2, SMMC-7721, HL7702, L02 cells, primary cell: 4401, 4403, 1204	Mice/BALB/cA nude	♂	8–10 weeks/?	?/daily for 21 days	Transplanted HepG2 cells 1 × 10^7^ cells/ml/0.2 ml	?	Immunohistochemistry (P53)

Darweish et al., 2014 [19]	Egypt	Chemopreventive and hepatoprotective effects of epigallocatechin gallate against hepatocellular carcinoma: role of heparan sulfate proteoglycans pathway	?/*Camellia sinensis*	?	Epigallocatechin gallate (EGCG)	?	HepG2 cell	Rats/Sprague Dawley	♂	?/180–200 g	?/twice for week for 16 days	Thioacetamide (200 mg/Kg b.w.)	?	H&E, immunohistochemistry (HSPGs), qRT-PCR (FGF-2), in serum (AST, albumin, and bilirubin), enzymatic determination (MDA, SOD, MPO, ELISA MMP-9, HSPGs, AFP, and syndecan-1)

Zheng et al., 2014 [20]	China/USA	Anticancer effects of Baicalein on hepatocellular carcinoma cells	?/*Scutellaria *sp.	Radix	Baicalein	Purchased	MIHAs, H22, BEL-7404, HepG2 cells	Mice/ICR	♂	?/18–22 g	Intraperitoneal/ daily for 12 days	Transplanted H22 2 × 10^5^ cells/ml/0.2 ml	Weight of tumors/experiment end	Immunohistochemistry (AKT, p-AKT Ser473, *β*-catenin, cyclin D1)

Fan et al., 2014 [21]	China	Luteoloside suppresses proliferation and metastasis of hepatocellular carcinoma cells by Inhibition of NLRP3 inflammasome	?/*Gentiana macrophylla *	?	Luteoloside	Purchased	Hep3B, SNU-449, Huh-7, SMMC-7721, MHCCLM3, MHCC97-H cell	Mice/BALB/cA nude	♂	6 weeks/?	Oral/daily for 4 weeks (subcutaneous) and 8 weeks (metastasis) groups	Transplanted SMMC-7721 2 × 10^6^ cells 7/0.2 ml	Volume of tumor (cm3)/every 3 to 4 days	H&E

Feng et al., 2014 [22]	China	Effect of grape procyanidins on tumor angiogenesis in liver cancer xenograft models	?	Seed	Proanthocyanidins (GPC)	?	?	Mice/ SPF Kunming	?	4–6 weeks/20 g	Intraperitoneal/ daily for 10 days	Transplanted H22 cells 1 × 10^6^ cell/ml/0.2 ml	?	Immunohistochemistry (VEGF and CD34), RT-PCR (VEGF and *β*-actin)

Zhang et al., 2013 [23]	China	Silybin-mediated inhibition of notch	?/*Silybum marianum *	?	Silybin (SIL)	?	HepG2 cells	Mice/athymic nude	♂	4–6 weeks/?	Intraperitoneal/5 times for week for 20 days	Transplanted HepG2 1 × 10^6^ cells/ml/?	Size of tumor/every 3 days	Western blot (Notch1, Hes1, RBP-J*κ*, survivin, cyclin D1, Bcl2, BAX, *β*-actin)

Yang et al., 2013 [24]	China	Antitumor effects of two extracts from *Oxytropis falcata* on hepatocellular carcinoma in vitro and in vivo	?/*Oxytropis falcata *	Whole plant	Flavonoids (FOF) 2′,4′-dihydroxychalcone	Ethanol extraction/?	SMMC-7721 cells	Mice/ICR	♂	5-6 weeks/18–22 g	Intragastric/?	Transplanted H22 cells 5 × 10^6^ cell/ml/0.2 ml	Tumor growth inhibitory ratio/experiment end	?

Yu et al., 2013 [25]	China	A study on the antitumor effect of total flavonoids from *Pteris multifida* poir in H22 tumor-bearing mice	?/*Pteris multifida *	Whole plant	Total flavonoids	Ethanol extraction/?	?	Mice/Kunming	1/2 ♂ and 1/2 ♀	?/18–22 g	Intraperitoneal/ daily for 10 days	Transplanted H22 cell 5 × 10^7^ cells/ml/0.3 ml	Tumor inhibition rate and organ index (spleen and thymus)/experiment end	ELISA (TNF-*α* and IL-2), enzymatic determination (T-AOC, MDA)

Xiang et al., 2013 [26]	China	Chemical composition of total flavonoids from* Salvia chinensia* Benth and their proapoptotic effect on hepatocellular carcinoma cells: potential roles of suppressing cellular NF-kB signaling	?/*Salvia chinensia *	Whole plant	Total flavonoids	Alcoholic extraction 95%/HPLC and chromatography in silica gel column	HepG2, Huh-7 HCC cells	Mice/Kunming	♂	?/18–22 g	Intraperitoneal/ daily for 10 days	Transplanted H22 cells 1 × 10^6^ cells/ml/?	Weight of tumor, organ index/experiment end	Automatic counting (red blood cells, hemoglobin, white blood cells, platelets), serum (AST, ALT, BUN, CK, uric acid), ELISA (caspase-3, caspase-8, caspase-9)

Hashimoto et al., 2014 [27]	Japan	Methylated-(3′′)-epigallocatechin gallate analog suppresses tumor growth in Huh7 hepatoma cells via inhibition of angiogenesis	?/*Camellia sinensis*	Green leaves	Polyphenolic, catechin, methylated-(3)-epigallocatechin gallate (MethylEGCG)	Purchased/HPLC purification	HUVECs, Huh7 cells	Mice/BALB/cA nude	♂	6 weeks/20 g	Intraperitoneal/ daily for 21 days	Transplanted Huh7 cells 5 × 10^6^ cell/ml/?	Size, volume of tumor/every week	Immunohistochemistry (CD31)

Saleem et al., 2013 [28]	India and Saudi Arabia	Anticancer potential of rhamnocitrin 40-b-D-galactopyranoside against N-diethylnitrosamine-induced hepatocellular carcinoma in rats	?/*Astragalus hamosus*	Leaves	Flavonol glycoside (rhamnocitrin 40-b-D-galactopyranoside RGP)	MeOH/H2O extraction/column Sephadex LH-20	?	Rats/Wistar	?	?/150–220 g	Intraperitoneal/ daily for 20 weeks	Single intraperitoneal dose of N-nitrosodiethylamine (NDEA) (200 mg/kg b.w.)	?	Serum (ALT, AST, ALP, total bilirubin, protein content, LPO), enzymatic determination (SOD, CAT, GPx, GST), H&E

Monga et al., 2013 [29]	India	Growth inhibition and apoptosis induction by (+)-Cyanidan-3-ol in hepatocellular carcinoma	Fabaceae/*Acacia catechu*	Heart wood	Cyanidan-3-ol (CD-3)	Ethanol extraction/HPLC	HepG2 cells	Mice/BALB/cA	♂	6-7 weeks/26-27 g	Oral/daily for 20 weeks	Single intraperitoneal dose of NDEA (200 mg/kg b.w.) and carbon tetrachloride (CCl4, 3 ml/kg b.w.) thrice a week for six weeks	Relative liver weight/experiment end	Serum (AST, ALT, ALP, *γ*-GT, TSA, LASA), enzymatic determination (MDA, SOD, CAT, GPx, GR, GST, T-SH, GSH, PrPr-SHs), H&E, immunohistochemistry (p53, p65, c-jun)

Zhao et al., 2013 [30]	China	Enhanced 5-fluorouracil cytotoxicity in high COX-2 expressing hepatocellular carcinoma cells by Wogonin via the PI3K/AKT pathway	?/*Scutellaria radix*	?	Wogonin 5,7-dihydroxy-8-methoxyflavone (WOG)	?	HepG2, SMMC-7721 cells	Mice/BALB/cA nude	♀	35–40 days/18–22 g	Intravenous/once every two days for 10 times	Transplanted SMMC-7721 cells 1 × 10^6^ cells/mouse/?	Tumor inhibitory ratio, weight, size, volume of tumor/experiment end	?

Wei et al., 2012 [31]	China	Antitumor and antiangiogenic effects of *Macrothelypteris viridifrons* and its constituents by HPLC-DAD/MS analysis	?/*Macrothelypteris viridifrons*	Air dried roots	2-(1,4-Dihydroxy-cyclohexyl)-5,7-dihydroxy-chromone-4′-O-glucoside; protoapigenin-4′-O-glucoside; 2-(1,2-dihydroxy-4-oxo-cyclohex-5-enyl)-5,7-dihydroxy-chromone; 5,7,2′,5′-tetrahydroxy-flavanone-2′-O-glucoside; protoapigenin; 2-(1,4-dihydroxy-cyclohexyl)-5,7-dihydroxy-chromone; 5,7,2′,5′-tetrahydroxy-flavanone-2′-O-6′′-O-acetylglucoside; 5,6-dihydro-6-methoxyprotoapigenone; quercetin-3-O-rutinose; protoapigenone; apigenin-4-O-glucoside; kaempferol-3-O-rutinose; kaempferol-3,7-di-O-rhamnoside; kaempferol-3-O-rhamnoside; apigenin-4-O-rhamnoside	?/HPLC-DAD/MS Amethyst C18-P column	HUVECs, H-22 cells	Mice/Kunming	♂	?/18–22 g	Intraperitoneal/ daily for 10 days	Transplanted H22 cells 2 × 10^6^ cells/ml/0.2 ml	Weight of tumor, tumor inhibitory ratio/experiment end	Serum (red blood cell, white blood cell, platelet and hemoglobin), BUN, CRE, AST and ALT, TSA, LASA, enzymatic determination (MDA, SOD, CAT, GPx, GR, GST, T-SH, GSH, PrPr-SHs), H&E, immunohistochemistry (CD34, VEGF)

Wu et al., 2011 [32]	Taiwan	Suppression of hepatitis B virus × protein-mediated tumorigenic effects by ursolic acid	Rubiaceae/*Morinda citrifolia*	Leaves	Ursolic acid and silymarin	?	Huh7, HepG2, Hep3B cells	Mice/nude	?	3 weeks/?	Intraperitoneal/?/8 weeks	Transplanted 6 2.2.15 cells 1 × 10^6^ cell/ml/0.1 ml	Diameters of tumor/experiment end	Serum (ALT, AST, BUN, CRE)

Wang et al., 2011 [33]	Taiwan	*Solanum nigrum* L. polyphenolic extract inhibits hepatocarcinoma cell growth by inducing G2/M phase arrest and apoptosis	?/*Solanum nigrum*	Whole plant	Total flavonoids	Water extract (SNWE)/?	HepG2 cells	Mice/Athymic nude	?	?	Intraperitoneal/?/35 days	Transplanted HepG2 cells 5 × 10^6^ cell/ml/0.4 ml	Volume of tumor/every week, final volume, wet weight of tumor/experiment end	?

Cai et al., 2011 [34]	China	Apigenin inhibits hepatoma cell growth through alteration of gene expression patterns	?	?	Apigenin	?	Huh7 cells	Mice/BALB/c nude	♀	5 weeks/16–18 g	Intraperitoneal/?/30 days	Transplanted Huh7 cells 2 × 10^6^ cell/ml/?	Number of tumors, diameters, wet weight of tumor/experiment end	H&E

Huang et al., 2010 [35]	China	Carbonyl reductase 1 as a novel target of (−)-epigallocatechin gallate against hepatocellular carcinoma	?/*Camellia sinensis*	?	Epigallocatechin gallate (EGCG)	?	Hep G2, Hep 3B, SMMC-7721 cells	Mice/?	?	?	Injected/?/15 days	Transplanted SMMC-7721 cells and Hep3B/?/?	Tumor growth, body weight/?	Serum (ALT, AST, LDH, CK-MBB, LDH, MDA, cTnT)

Liang et al., 2010 [36]	China and USA	Green tea catechins augment the antitumor activity of doxorubicin in an *in vivo* mouse model for chemoresistant liver cancer	?/*Camellia sinensis*	?	Catechin and epigallocatechin-3-gallate	?	BEL-7404, BEL-7404/DOX cells	Mice/ BALB/cA nude	♂ and ??	4-5 weeks/13–17 g	Intraperitoneal/?/33 days	Transplanted BEL-7404/DOX HCC cells 5 × 10^7^ cell/ml/0.2 ml	Dimensions of tumor, volume of tumor/every 2 days, tumor growth, body weight/experiment end	Fluorospectrophotometry (DOX), immunohistochemistry, RT-PCR (MDR1)

Zhou et al., 2010 [37]	China	Inhibition of hepatoma 22 tumor by liquiritigenin	?/*Glycyrrhiza glabra*	?	Liquiritigenin (LQ)	Purchased	?	Mice/ICR	♂	?/20–22 g	Intragastric/?/15 days	Transplanted H22 cells 10 × 10^6^ cell/ml/0.2 ml	Size, volume of tumor, body weight/every 3 days, Inhibition ratio of tumor growth, weight of tumor, organ index/experiment end	H&E, electron microscopy (tumor ultrastructure), enzymatic determination (MDA)

Yang et al., 2009 [38]	China	Antiproliferative efficacy of icariin on HepG2 hepatoma and its possible mechanism of action	?/*Herba epimedii *	?	Icariin (5-hydroxy 4-methoxy 8-isopentenyl 3-O-*α*-rhamnosyl 7-O-*β*-glucosyl flavone)	Purchased	HepG2 cells	Mice/NMRI nude	♂	?	Oral/28 weeks	Transplanted HepG2 cells 1 × 10^7^ cell/ml/?	Volume of tumor, tumor growth inhibition/twice per week	Immunohistochemistry (CD4, CD8, CD19)

Zhao et al., 2010 [39]	China	Synergistic effect of 5-fluorouracil and the flavonoid Oroxylin A on HepG2 human hepatocellular carcinoma and on H22 transplanted mice	?/*Scutellariae Radix*	?	Oroxylin A	?	HepG2 cells	Mice/Kunming	♂	?/18–22 g	Oral/?/7 days	Transplanted H22 cells 5 × 10^6^ cells/ml/0.9 ml	Volume of tumor/experiment end	?

Cui et al., 2009 [40]	USA	Effects and mechanisms of Silibinin on human hepatocellular carcinoma xenografts in nude mice	?/*Silybum marianum*	?	Silibinin	?	HuH7 cells	Mice/nude	?	?	Oral/?/5 weeks	Transplanted HuH7 cells 5 × 10^6^ cell/ml/0.25 ml	Volumes of tumor/every week, weight of tumor/experiment end	Immunoprecipitation (AFP, PTEN, binding interaction between p21, p27 with CDK4, binding of DP1 with E2F1), Western blotting (Ki-67, p21, p27, E2F1, CDK4, p-Rb, caspase-3, caspase-9, PTEN, AC-H3, AC-H4, p-AKT, p-survivin and p-ERK, Plk1, Chk1, SOD1), ELISA (NF-kB)

Umarani et al., 2008 [41]	India	Protective effect of *Kalpaamruthaa* in combating the oxidative stress posed by aflatoxin B1-induced hepatocellular carcinoma with special reference to flavonoid structure-activity relationship	Anacardiaceae/ *Semecarpus anacardium*	Fruit	Flavonoids	Dried and powdered fruit/?	?	Rats/?	♂	8–10 weeks/120–150 g	Oral/?/28 days	Intraperitoneal dose of AFB1 (2 mg/kg b.w.)	?	Serum (protein, LPO, lipid peroxides, G6PD, vitamin E, vitamin C, uric acid), enzymatic determination (SOD, CAT, GPx, GR)

Miura et al., 2007 [42]	Japan	Effect of apple polyphenol extract on hepatoma proliferation and invasion in culture and on tumor growth, metastasis, abnormal lipoprotein profiles in hepatoma-bearing rats	?/*Malusdomestica*	Unripe apples	Apple polyphenol extract (APE)	Purchased	AH109A cells	Rats/Donryu	♂	5 weeks/?	Oral/?/21 days	Transplanted AH109A cells 1 × 10^7^ cells/ml/0.5 ml	Size of tumor/every day	Serum (T-Ch, HDL-Ch), LPO, fecal steroid, AI

Selvendiran et al., 2006 [43]	Japan	Luteolin promotes degradation in signal transducer and activator of transcription 3 in human hepatoma cells: an implication for the antitumor potential of flavonoids	?	Seeds	Luteolin	?/HPLC	HepG2, HLF, HAK-1B, IMR-32 cells	Mice/BALB/cA nude	♂	5 weeks/?	Oral/?/6 weeks	Transplanted HAK-1B cells 1 × 10^7^ cell/ml/?	Size, volume (mm3) of tumor/weekly	Immunoblotting (Tyr705-P-STAT3)

Nishikawa et al., 2006 [44]	Japan	A green tea polyphenol, epigallocatechin-3-gallate, induces apoptosis of human hepatocellular carcinoma, possibly through inhibition of Bcl-2 family proteins	?/*Camellia sinensis *	?	Epigallocatechin-3-gallate	?	HLE, HepG2, HuH-7, PLC/PRF/5 cells	Mice/BALB/cA nude	♂	28 weeks/?	Oral/?/25 days	Transplanted HLE cells 1 × 10^6^ cell/ml/?	Volume of tumor/daily	TUNEL, immunohistochemistry (Bcl-2a, Bcl-xl)

Premalatha and Sachdanandam, 1999 [45]	India	*Semecarpus anacardium* L. nut extract administration induces the in vivo antioxidant defense system in aflatoxin B1 mediated hepatocellular carcinoma	?/*Semecarpus anacardium* L	Nuts	Total flavonoids	Purchased	?	Rat/Wistar	♂	?/100 g	Oral/?/14 days	Single intraperitoneal dose of AFB1 (2 mg/kg b.w.)	Weight of liver and kidney/experiment end	Total protein, enzymatic determination (GSH, uric acid, vitamin E, vitamin C, CYP, T-SH, NPSH)

Nishida et al., 1994 [46]	Japan	Inhibitory effects of (−)-epigallocatechin gallate on spontaneous hepatoma in C3H/HeNCrj mice and human hepatoma-derived PLC/PRF/5 cells	?/*Camellia sinensis*	?	Epigallocatechin gallate (EGCG)	?	PLC/PRF5 cells	Mice/C3H/HenCrj	♂	8 weeks/?	Oral/?/65 weeks	Spontaneous hepatocarcinogenesis	# of tumor, diameter of liver/experiment end	H&E, albumin, bilirubin, GPT, *γ*-GT, total cholesterol

Zhang et al., 2002 [47]	Japan	Effects of dietary powdered green tea and theanine on tumor growth and endogenous hyperlipidemia in hepatoma-bearing rats	?/*Camellia sinensis*	?	?	?	?	Rat/Donryu	♂	4 weeks/?	Oral/?/14 days	Transplanted AH109A cells 5 × 10^5^ cell/ml/0.5 ml	Size, volume, diameter of tumor, radius tumor/every day, weight of tumor/experiment end	Precipitation method (HDL, LDL, VLDL), enzymatic determination (T-Ch and HDL-ChM, TBARS), fecal extraction (neutral sterol, bile acid)

Klaunig, 1992 [48]	USA	Chemopreventive effects of green tea components on hepatic carcinogenesis	?/*Camellia sinensis*	?	Epicatechin, epicatechin gallate, epigallocatechin, epigallocatechin gallate	Methanol extraction/steel column with silicic acid	Primary cells mouse hepatocytes	Rat/B_6_C_3_F_1_	♂	?	Oral/?/28 weeks	Single intraperitoneal dose NDEA (90 mg/kg b.w.)	?	H&E

?: not specified; ♀: female; ♂: male; b.w.: body weight; ELISA: enzyme-linked immunosorbent assay; H&E: hematoxylin and eosin histological staining; qRT-PCR: real-time quantitative reverse transcription polymerase chain reaction; TUNEL: terminal deoxynucleotidyl transferase dUTP nick end labeling; AC-H3: acetilated histone H3; AC-H4: acetilated histone H4; AKT: protein kinase B; P-AKT_Ser473_: phosphorylated AKT serine 473; Bcl-2: B-cell lymphoma 2; BAX: Bcl-2 associated X protein; CD4: *cluster of cuadruple differentiation; CD8:* cluster of differentiation 8; CD19: cluster of differentiation 19; CD31: cluster of differentiation 31; CD34: hematopoietic progenitor cell antigen CD34; CDK4: cyclin-dependent kinase 4; P21: cyclin-dependent kinase inhibitor 1; P27: cyclin-dependent kinase inhibitor 1B (CDKN1B); P-ERK: phosphorylated extracellular signal-regulated kinases; Plk1: polo-like kinase 1; Chk1: checkpoint kinase 1; cTnT: cardiac troponin T; CYP: cytochrome P450; DOX: doxorubicin; DP1: transcriptional factor DP1; E2F1: transcriptional factor E2F1; Hes1: transcription factor HES1; FGF-2: fibroblast growth factor; VEGF: vascular endothelial growth factor; IL-2: interleukin 2; Ki-67: Ki-67 antigen; MDR1: multidrug resistance protein 1; MMP9: matrix metallopeptidase 9; NF-*κ*B: nuclear factor kappa-light-chain-enhancer of activated B cells; Notch 1: Notch homolog 1; P53: P53 protein; P65: nuclear factor NF-kappa-B p65 subunit; P-Rb: retinoblastoma protein; PTEN: phosphatase and tensin homolog; RBP-jk: recombining binding protein suppressor of hairless; STAT3: signal transducer and activator of transcription 3; TNF-*α*: tumor necrosis factor-alpha; Tyr 705-P-STAT3: phosphorylated signal transducer and activator of transcription 3; CAT: catalase; LPO: lipid peroxidation; MDA: malondialdehyde; SOD: superoxide anion; SOD1: superoxide dismutase 1; T-AOC: total antioxidant capacity; TBARS: thiobarbituric acid reactive substances; AFP: alpha-fetoprotein; AI: atherogenic index; ALP: alkaline phosphatase; ALT: alanine aminotransferase; AST: aspartate aminotransferase; BUN: blood urea nitrogen; CK-MB: creatinine kinase-MB; CK: creatinine kinase; G6PD: glucose-6-phosphate dehydrogenase; GPx: glutathione peroxidase; GPT: glutamic-pyruvic transaminase; *γ*-GT: gamma-glutamyl transferase; GR: glutathione reductase; GSH: reduced glutathione; GST: glutathione transferase; HDL-Ch: high-density lipoprotein cholesterol; LDL: low-density lipoprotein cholesterol; VLDL: very low-density lipoprotein cholesterol; HSPGs: heparan sulfate proteoglycans; LASA: lipid associated sialic acid; LDH: lactate dehydrogenase; MPO: hepatic myeloperoxidase; PrPr-SHs: protein thiols; TBARS: thiobarbituric acid reactive substances; T-Ch: triglyceride; TSA: total sialic acid; T-SH: total thiol; NPSH: nonprotein thiol.

**Table 2:** ARRIVE of the studies using flavonoids for the treatment of hepatocellular carcinoma in murine models.

	Reference																																		%
	[15]	[16]	[17]	[18]	[19]	[20]	[21]	[22]	[23]	[24]	[25]	[26]	[27]	[28]	[29]	[30]	[31]	[32]	[33]	[34]	[35]	[36]	[37]	[38]	[39]	[40]	[41]	[42]	[43]	[44]	[45]	[46]	[47]	[48]	
**Title**																																			
Accurate and concise a description of the content of the article	X	X	X	X	X	X	X	X	X	X	X	X	X	X	X	X	X	X	X	X	X	X	X	X	X	X	X	X	X	X	X	X	X	X	100%
**Abstract**																																			
Summary of the background, objectives, methods, principal findings, and conclusions	X	X	X	X	X	X	X	X	X	X	X	X	X	X	X	X	X	X	X	X	X	X	X	X	X	X	X	X	X	X	X	X	X	X	100%
**Introduction**																																			
Background	X	X	X	X	X	X	X	X	X	X	X	X	X	X	X	X	X	X	X	X	X	X	X	X	X	X	X	X	X	X	X	X	X	X	100%
Objectives	X	X	X	X	X	X	X	X	X	X	X	X	X	X	X	X	X	X	X	X	X	X	X	X	X	X	X	X	X	X	X	X	X	X	100%
**Materials and methods**																																			
*Ethical statement*																																			
Indicate the nature of the ethical review permissions, relevant licenses	X	X	X	X	X	X	X	X	X	X			X		X	X	X	X		X		X	X		X		X	X	X						64.71%
*Study design*																																			
Number of experimental and control groups	X	X	X	X	X	X	X	X	X	X	X	X	X	X	X	X	X	X	X	X	X		X	X	X	X	X	X	X						82.35%
Any steps taken to minimize the effects of subjective bias when allocating animals to treatment	X	X		X		X	X	X	X		X	X	X	X	X	X	X		X			X	X	X			X	X							58.82%
The experimental unit	X	X	X	X	X	X	X	X	X	X	X	X	X	X	X	X	X										X								52.94%
*Experimental procedures*																																			
Doses	X	X	X	X	X	X	X	X	X	X	X	X	X	X	X	X	X	X	X	X	X	X	X	X	X	X	X	X	X	X	X	X	X	X	100%
Method of administration	X	X	X		X	X	X	X	X	X	X	X	X	X	X	X	X	X		X	X			X			X	X							64.71%
Time of day	X	X	X	X	X	X	X	X	X	X	X	X	X	X	X	X	X	X		X															55.88%
*Experimental animals*																																			
Origin of animal	X			X				X	X	X	X	X	X	X	X	X	X	X	X	X	X	X	X	X	X	X	X	X	X	X	X	X	X	X	85.29%
Species	X	X	X	X	X	X	X	X	X	X	X	X	X	X	X	X	X	X	X	X	X	X	X	X	X	X	X	X	X	X	X	X	X	X	100%
Sex	X	X	X	X	X	X	X		X	X	X	X	X		X	X	X			X	X	X	X	X			X	X							64.71%
Developmental stage	X		X	X			X	X	X	X			X		X	X		X	X	X		X			X		X	X							50%
Weight	X	X			X	X		X		X	X	X	X	X	X	X	X			X		X	X		X		X								52.94%
*Housing and husbandry*																																			
Housing	X	X	X		X			X		X				X	X		X		X				X		X		X	X							41.17%
Husbandry conditions	X		X	X	X	X		X		X		X		X	X		X		X			X	X		X		X	X							50%
*Sample size*																																			
Specify the total number of animals used in each experiment	X	X	X	X	X	X	X	X	X	X	X	X	X	X	X	X	X	X	X					X				X							61.76%
Explain how the number of animals was decided																																			0%
*Allocating animals to experimental groups*																																			
How animals were allocated to experimental group (AZAR)	X	X	X	X		X	X	X	X		X	X			X	X	X	X	X	X			X	X				X							55.88%
*Experimental outcomes*																																			
Clearly define the primary and secondary experimental outcomes assessed	X		X		X		X					X		X	X		X	X	X	X	X		X	X			X	X							47.05%
*Statistical methods*																																			
Provide details of the statistical methods used for each analysis	X	X	X	X	X	X	X	X	X	X	X	X	X	X	X	X	X	X	X	X			X	X			X	X							70.58%
Specify the unit of analysis for each dataset	X	X	X	X	X	X	X	X	X	X	X	X	X	X	X	X	X	X	X	X			X	X			X	X							70.58%
Describe any methods used to assess whether the data met the assumptions of the statistical approach		X							X			X				X	X	X	X	X			X	X			X	X							35.29%
**Results**																																			
*Baseline data*																																			
For each experimental group, report relevant characteristics and health status of animals before treatment or testing	X		X					X							X								X												14.70%
*Numbers analyzed*																																			
Report the number of animals in each group included in each analysis	X	X			X		X		X	X			X	X	X		X	X	X				X	X			X								44.11%
If any animals or data were not included in the analysis, explain why																																			0%
*Outcomes and estimation*																																			
Report the results for each analysis carried out	X	X	X	X	X	X	X	X	X	X	X	X	X	X	X	X	X	X	X	X	X	X	X	X	X	X	X	X	X	X	X	X	X	X	100%
*Adverse events*																																			
Give details of all important adverse events in each experimental group																								X											2.94%
Describe any modifications to the experimental protocols made to reduce adverse events																	X																		2.94%
**Discussion**																																			
*Interpretation/* *scientific implications*																																			
Interpret the results, taking into account the study objectives and hypotheses, current theory, and other relevant studies in the literature	X	X	X	X	X	X	X	X	X	X	X	X	X	X	X	X	X	X	X	X	X	X	X	X	X	X	X	X	X	X	X	X	X	X	100%
Comment on the study limitations including any potential sources of bias																								X											2.94%
*Generalizability/* *translation*																																			
Comment on whether and how the findings of this study are likely to translate to other species or systems, including any relevance to human biology						X	X			X			X												X		X								17.64%
*Funding*																																			
List all funding sources	X	X	X	X		X	X	X	X			X	X	X	X		X	X	X	X	X	X	X	X	X			X	X		X	X			73.52%

%	80%	68.57%	68.57%	62.85%	62.85%	65.71%	68.57%	71.42%	68.57%	71.42%	57.14%	68.57%	68.57%	65.71%	80.00%	65.71%	80.00%	62.85%	62.85%	65.71%	40.00%	45.71%	68.57%	65.71%	48.57%	28.57%	71.42%	68.57%	34.28%	25.71%	28.57%	28.57%	25.71%	25.71%	

## Supplementary Materials

Supplementary Materials Table S1: descriptors used for advanced search in PubMed and Scopus.
